# Supplementary material for: Earthworms act as biochemical reactors to convert labile plant compounds into stabilized soil microbial necromass
Source: Commun Biol. 2019 Nov 28;2:441. doi: 10.1038/s42003-019-0684-z (PMC6883063; doi:10.1038/s42003-019-0684-z)
Supplement: Supplementary file 1 — Supplementary Information [file 42003_2019_684_MOESM1_ESM.pdf]

Angst et al.: Earthworms as biochemical reactors – converting labile plant compounds to stabilised soil microbial necromass

Supplementary Material

Supplementary Figures

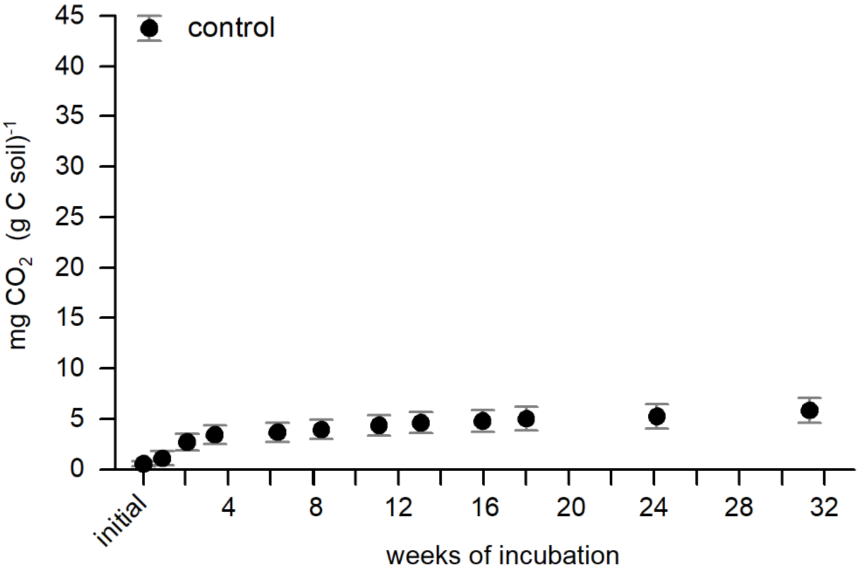

Supplementary Fig. 1. Cumulative heterotrophic respiration from the control treatments without any addition (neither plant material nor earthworms) during the experimental period. The markers represent arithmetic means of three replicates. Error bars indicate standard errors.

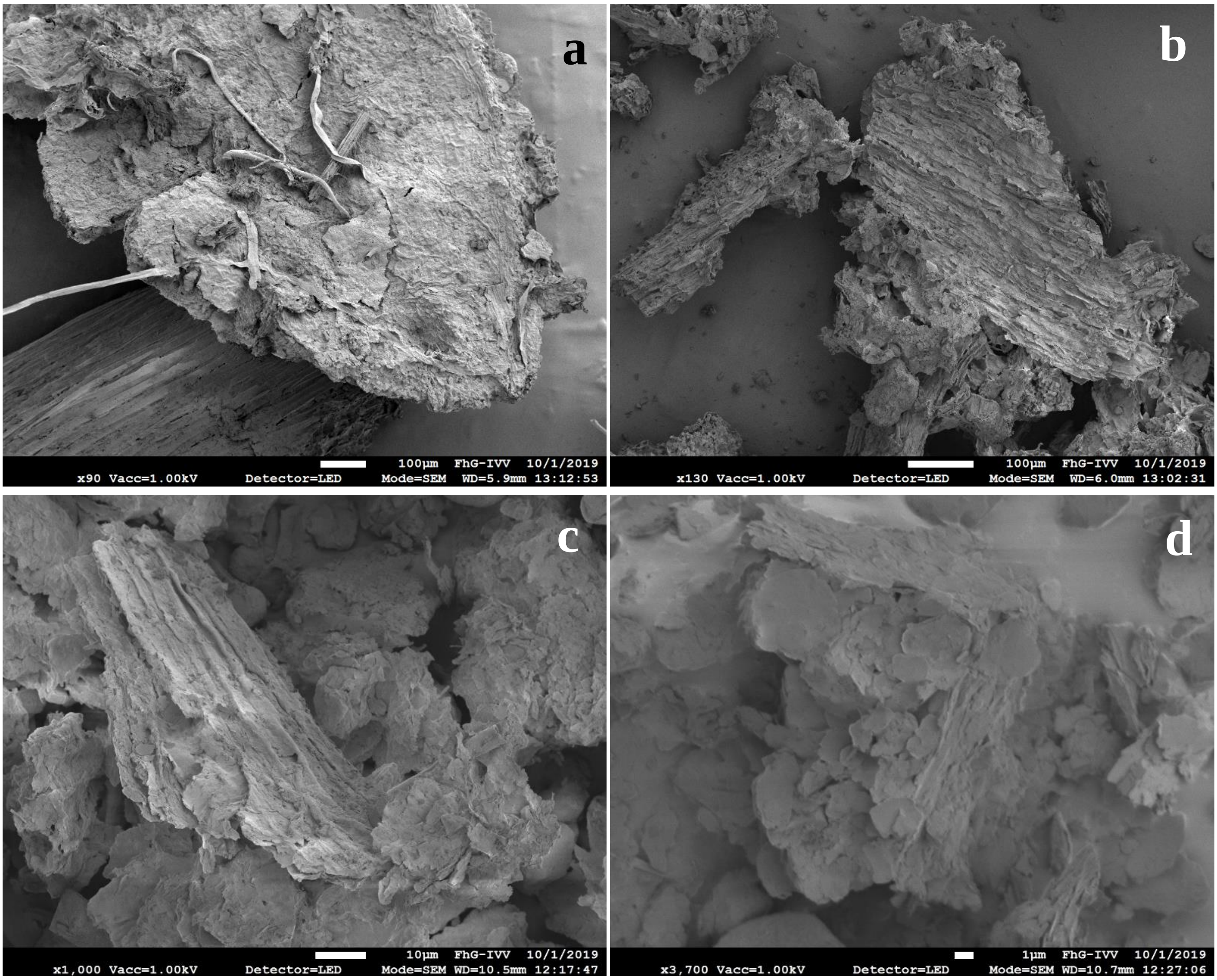

Supplementary Fig. 2. Scanning electron micrographs (Jeol 7200 F, Germany) of selected soil organic matter fractions. a) fPOM with visible fungal hyphae on the surface of the decaying plant residue, b) oPOMmacro with clearly visible plant cell structures of the plant residues, c) oPOMmicro with a larger amount of amorphous organic particles besides still visible cell structures and d) clay (macro and micro))

Supplementary Tables

Supplementary Table 1. Weight and organic C of soil fractions from the control treatments without any addition (neither plant material nor earthworms). Values represent arithmetic means of three replicates. SE = standard deviation.

**fraction**	**weight** [mg fraction (g bulk soil)$^{-1}$]	**SE**	**organic C** [%C fraction (C bulk soil)$^{-1}$]	**SE**
**fPOM**	1.1	0.3	10.1	3.3
**oPOM**	1.1	0.1	8.4	2.1
**clay**	3.3	0.9	12.6	0.8
**oPOMmicro**	8.3	1.5	4.8	1.2
**claymicro**	155.7	2.5	44.9	2.2

Supplementary Table 2. Basic soil properties of the initial Soil used for the incubation.

**Property**	**Value**
pH	5.8
soil N content [mg g^{-1}]	0.3
bulk density [g cm^{-3}]	1.2
