# Supplementary material for: Earthworms act as biochemical reactors to convert labile plant compounds into stabilized soil microbial necromass
Source: Commun Biol. 2019 Nov 28;2:441. doi: 10.1038/s42003-019-0684-z (PMC6883063; doi:10.1038/s42003-019-0684-z)
Supplement: Supplementary file 2 — Description of Additional Supplementary Files [file 42003_2019_684_MOESM2_ESM.pdf]

Description of additional supplementary files

Supplementary Data 1

This file includes the raw data for the following variables listed for each fraction (fPOM, $oPOM_{macro}$, $clay_{macro}$, $oPOM_{micro}$, $clay_{micro}$) and each of the three replicates for treatments with or without the presence of earthworms:

- weight mass [mg g $soil^{-1}$]
- proportion of carbon [%C fraction bulk soil C^{-1}]
- NMR mixing model integration regions [%]
- Plant-derived lipids [mg g C^{-1}]
- Sum of amino sugars [mg g C^{-1}]

The last three columns of the file display the bulk soil carbon contents of the initial soil used for the incubation and for the treatments with or without earthworms after the incubation for three replicates.

Supplementary Data 2

This file includes the values for cumulative heterotrophic respiration during the experimental period for treatments with and without earthworms in mg CO_2 g soil C^{-1}.
