# Supplementary material for: Earthworms act as biochemical reactors to convert labile plant compounds into stabilized soil microbial necromass
Source: Commun Biol. 2019 Nov 28;2:441. doi: 10.1038/s42003-019-0684-z (PMC6883063; doi:10.1038/s42003-019-0684-z)
Supplement: Supplementary file 5 — Reporting Summary [file 42003_2019_684_MOESM5_ESM.pdf]

nature research

Corresponding author(s): Gerrit Angst

Last updated by author(s): Oct 2, 2019

# Reporting Summary

Nature Research wishes to improve the reproducibility of the work that we publish. This form provides structure for consistency and transparency in reporting. For further information on Nature Research policies, see Authors & Referees and the Editorial Policy Checklist.

## Statistics

For all statistical analyses, confirm that the following items are present in the figure legend, table legend, main text, or Methods section.

| n/a | Confirmed | |
|---|---|---|
| ☐ | ☒ | The exact sample size (*n*) for each experimental group/condition, given as a discrete number and unit of measurement |
| ☐ | ☒ | A statement on whether measurements were taken from distinct samples or whether the same sample was measured repeatedly |
| ☐ | ☒ | The statistical test(s) used AND whether they are one- or two-sided<br>*Only common tests should be described solely by name; describe more complex techniques in the Methods section.* |
| ☒ | ☐ | A description of all covariates tested |
| ☐ | ☒ | A description of any assumptions or corrections, such as tests of normality and adjustment for multiple comparisons |
| ☐ | ☒ | A full description of the statistical parameters including central tendency (e.g. means) or other basic estimates (e.g. regression coefficient) AND variation (e.g. standard deviation) or associated estimates of uncertainty (e.g. confidence intervals) |
| ☐ | ☒ | For null hypothesis testing, the test statistic (e.g. $F$, $t$, $r$) with confidence intervals, effect sizes, degrees of freedom and $P$ value noted<br>*Give P values as exact values whenever suitable.* |
| ☒ | ☐ | For Bayesian analysis, information on the choice of priors and Markov chain Monte Carlo settings |
| ☒ | ☐ | For hierarchical and complex designs, identification of the appropriate level for tests and full reporting of outcomes |
| ☐ | ☒ | Estimates of effect sizes (e.g. Cohen's $d$, Pearson's $r$), indicating how they were calculated |

*Our web collection on statistics for biologists contains articles on many of the points above.*

## Software and code

Policy information about availability of computer code

| | |
|---|---|
| Data collection | Chemstation (B.04.03), XCalibur (3.1), MassHunter (B.07.04.2260), Chromeleon Version 7.1.3.2425 |
| Data analysis | R for Windows version 3.3.2; Microsoft Excel 2016 for Windows |

For manuscripts utilizing custom algorithms or software that are central to the research but not yet described in published literature, software must be made available to editors/reviewers. We strongly encourage code deposition in a community repository (e.g. GitHub). See the Nature Research guidelines for submitting code & software for further information.

## Data

Policy information about availability of data

All manuscripts must include a data availability statement. This statement should provide the following information, where applicable:

- Accession codes, unique identifiers, or web links for publicly available datasets
- A list of figures that have associated raw data
- A description of any restrictions on data availability

We attached all the raw data the manuscript is based on in a supplementary table.

# Field-specific reporting

Please select the one below that is the best fit for your research. If you are not sure, read the appropriate sections before making your selection.

☐ Life sciences ☐ Behavioural & social sciences ☒ Ecological, evolutionary & environmental sciences

For a reference copy of the document with all sections, see nature.com/documents/nr-reporting-summary-flat.pdf

# Ecological, evolutionary & environmental sciences study design

All studies must disclose on these points even when the disclosure is negative.

| | |
|---|---|
| Study description | We sampled soil in the field on the profile walls of three soil pits, randomly distributed accross our study site to account for spatial variability. These samples were later combined to a composite sample with which our incubation experiment was set up. The incubation experiment consisted of three treatments: control treatment (soil without any amendments), soil amended with leaves and roots, and soil amended with leaves and roots with the addition of earthworms. Each treatment was replicated five times. |
| Research sample | see above; five replicates per treatment. Earthworms added were Lumbricus rubellus, Hoffmeister (one specimen) and Aporrectodea caliginosa, Savigny (two specimens). The earthworm density and species in our experiment simulated the conditions in natural systems. Due to the labor intensive laboratory work (i.e., physical fractionation, elemental analysis, extraction of lipids and amino sugars, analysis of the extracts on GC/MS and GC/FID and performance of 13C nuclear magnetic resonance spectroscopy), we analysed three replicates per treatment further, which is still a reasonable size for a good representation of the mean. |
| Sampling strategy | The chosen sample size of five replicates per treatment in our incubation experiment assured a sufficiently high number of replicates to make sound statements even if some of the replicates had to be cancelled (e.g., due to a high earthworm mortality), which, however, was not the case. The eventually processed number of three replicates was a balancing of the labor intensive analyses we performed and the ability to make statistically sound statements. |
| Data collection | During the incubation, heterotrophic soil respiration was measured via titrating back sodium-hydroxide in small vials not reacted with evolving CO2 after a fixed time in closed jars. These measurements were done by Gerrit Angst and Veronika Jilkova. Soils from the incubation were dried, sieved and physically fractionated using a combined density, aggregate, and particle-size fractionation. The soil fractions containing most carbon were further processed. Lipids were extracted from these fractions using a sequential extraction procedure, including solvent extraction and base hydrolysis. The extracts were filtered, derivatised, and measured on a GC/MS for qualification (aided by a mass spectral library) and on a GC/FID for quantification (via comparison to an internal standard). Amino sugars were extracted from the soil fractions using acid hydrolysis. The extracts were filtered, derivatised and measured on a GC/FID (qualified and quantified based on an external standard containing the target compounds). The physical fractionations, extractions, and measurements were performed by Gerrit Angst. The soil fractions were also analysed by 13C nuclear magnetic resonance spectroscopy. These measurements were performed by Carsten W. Mueller and Isabel Prater. |
| Timing and spatial scale | The incubation experiment lasted 33 weeks, which corresponds to the time earthworms need to consume all the added plant material. Except for the heterotrophic respiration data, which was repeatedly collected during the incubation (at short intervals in the beginning and at longer intervals with increasing experiment duration, due to a continuous depletion of available substrate for microbial respiration after the initiation of the experiment), all the data were collected after the end of the incubation. |
| Data exclusions | No data was excluded from the analyses |
| Reproducibility | Some replicates were fractionated or extracted and measured twice. The obtained values were close to each other, indicating a good reproducibility of the methods used. |
| Randomization | Samples were grouped according to the respective treatment (see above). The microcosms for the different treatments were randomly selected. |
| Blinding | Since we performed an experiment with earthworms, blinding was not relevant. |

Did the study involve field work? ☒ Yes ☐ No

## Field work, collection and transport

| | |
|---|---|
| Field conditions | around 20°C, partly clouded, well-aerated soil conditions |
| Location | 48°54'29.8"N 14°28'21.9"E; 405 m asl |
| Access and import/export | The study site was freely accessible |
| Disturbance | We refilled the dug soil pits after sampling |

# Reporting for specific materials, systems and methods

We require information from authors about some types of materials, experimental systems and methods used in many studies. Here, indicate whether each material, system or method listed is relevant to your study. If you are not sure if a list item applies to your research, read the appropriate section before selecting a response.

## Materials & experimental systems

| n/a | Involved in the study |
|---|---|
| ☒ | ☐ Antibodies |
| ☒ | ☐ Eukaryotic cell lines |
| ☒ | ☐ Palaeontology |
| ☐ | ☒ Animals and other organisms |
| ☒ | ☐ Human research participants |
| ☒ | ☐ Clinical data |

## Methods

| n/a | Involved in the study |
|---|---|
| ☒ | ☐ ChIP-seq |
| ☒ | ☐ Flow cytometry |
| ☒ | ☐ MRI-based neuroimaging |

# Animals and other organisms

Policy information about studies involving animals; ARRIVE guidelines recommended for reporting animal research

| | |
|---|---|
| Laboratory animals | no lab animals involved |
| Wild animals | Earthworms, Lumbricus rubellus, Hoffmeister and Aporrectodea caliginosa, Savigny were collected by hand. They were transported to the lab in aerated plastic containers containing wet cloth. The earthworms were released back into nature after the experiment. |
| Field-collected samples | Field moist soil samples were transferred to the laboratory in plastic bags. The soils were dried and used for the incubation experiment. |
| Ethics oversight | no ethical approval or guidance was required |

Note that full information on the approval of the study protocol must also be provided in the manuscript.
